# Dynamic Susceptibility Perfusion Imaging for Differentiating Progressive Disease from Pseudoprogression in Diffuse Glioma Molecular Subtypes

**DOI:** 10.3390/jcm10040598

**Published:** 2021-02-05

**Authors:** Vivien Richter, Uwe Klose, Benjamin Bender, Katharina Rabehl, Marco Skardelly, Jens Schittenhelm, Ghazaleh Tabatabai, Johann-Martin Hempel, Ulrike Ernemann, Cornelia Brendle

**Affiliations:** 1 Department of Neuroradiology, Universitätsklinikum Tübingen, 72070 Tübingen, Germany; uwe.klose@med.uni-tuebingen.de (U.K.); benjamin.bender@med.uni-tuebingen.de (B.B.); k.rabehl@web.de (K.R.); Johann-Martin.Hempel@med.uni-tuebingen.de (J.-M.H.); ulrike.ernemann@med.uni-tuebingen.de (U.E.); cornelia.brendle@med.uni-tuebingen.de (C.B.); 2 Department of Neurosurgery, Universitätsklinikum Tübingen, 72070 Tübingen, Germany; marco.skardelly@med.uni-tuebingen.de; 3 Department of Neuropathology, Universitätsklinikum Tübingen, 72070 Tübingen, Germany; jens.schittenhelm@med.uni-tuebingen.de; 4 Interdisciplinary Section of Neurooncology, Department of Neurology, Universitätsklinikum Tübingen, 72070 Tübingen, Germany; ghazaleh.tabatabai@med.uni-tuebingen.de

**Keywords:** diffuse glioma, dynamic susceptibility perfusion imaging, DSC-MRI, pseudoprogression, progressive disease, DTM

## Abstract

Rationale and Objectives: Advanced adjuvant therapy of diffuse gliomas can result in equivocal findings in follow-up imaging. We aimed to assess the additional value of dynamic susceptibility perfusion imaging in the differentiation of progressive disease (PD) from pseudoprogression (PsP) in different molecular glioma subtypes. Materials and Methods: 89 patients with treated diffuse glioma with different molecular subtypes (IDH wild type (Astro-IDH^wt^), IDH mutant astrocytomas (Astro-IDH^mut^) and oligodendrogliomas), and tumor-suspect lesions on post-treatment follow-up imaging were classified into two outcome groups (PD or PsP) retrospectively by histopathology or clinical follow-up. The relative cerebral blood volume (rCBV) was assessed in the tumor-suspect FLAIR and contrast-enhancing (CE) lesions. We analyzed how a multilevel classification using a molecular subtype, the presence of a CE lesion, and two rCBV histogram parameters performed for PD prediction compared with a decision tree model (DTM) using additional rCBV parameters. Results: The PD rate was 69% in the whole cohort, 86% in Astro-IDH^wt^, 52% in Astro-IDH^mut^, and 55% in oligodendrogliomas. In the presence of a CE lesion, the PD rate was higher with 82%, 94%, 59%, and 88%, respectively; if there was no CE lesion, however, the PD rate was only 44%, 60%, 40%, and 33%, respectively. The additional use of the rCBV parameters in the DTM yielded a prediction accuracy for PD of 99%, 100%, 93%, and 95%, respectively. Conclusion: Utilizing combined information about the molecular tumor type, the presence or absence of CE lesions and rCBV parameters increases PD prediction accuracy in diffuse glioma.

## 1. Introduction

The differentiation of diffuse gliomas by their histologic and molecular characteristics enables targeted adjuvant therapeutic management and a better estimation of prognosis. However, classical MR imaging tools such as T2 signal and contrast enhancement (CE) are often insufficient for the initial differentiation between tumor subtypes and in the post-therapeutic evaluation. Therapy-related changes include radiation necrosis (RN), pseudoprogression (PsP), and pseudoresponse (PR) [1]. PR can be detected as regressing edema and CE in the context of anti-VEGF therapy. In RN, the intense local tissue changes such as necrosis are radiotherapy (RT)-associated with a longer delay of up to 12 months and usually do not completely subside. In PsP, the increasing edema and disrupted blood-brain barrier in the radiation field is facilitated by the additional chemotherapy, may appear within the first 3–6 months after radiochemotherapy (RCT), and will regress over time. While PsP and RN are often confused, the latter should be reserved for persisting but stable lesions in patients without preceding chemotherapy [2]. The diagnosis of RN and PsP require follow-up imaging and cannot be differentiated reliably at the first imaging from progressive disease (PD) by conventional magnetic resonance imaging (MRI) [2,3]. The Response Assessment in Neuro-Oncology (RANO) group currently recommends clinical and/or imaging follow-ups to differentiate PD from PsP [4,5,6]. However, to detect PD earlier in order to enable a timely change in the therapeutical approach, it would be desirable to define imaging parameters to differentiate between PD and PsP. Dynamic susceptibility perfusion imaging (DSC-MRI) is a robust functional imaging tool assessing elevated vasculature in brain tumors by quantifying the contrast agent influx [7]. The neo-angiogenesis in glioma varies depending on the molecular characteristics, and isocitrate dehydrogenase (IDH) mutation status correlates with regional cerebral blood volume (rCBV) parameters [8]. As the presence of an IDH mutation correlates with better prognoses and better response to therapy [9], it is important to also tailor the diagnostics on the molecular subgroups. DSC-MRI´s utility to differentiate PsP and PD has been shown in prior studies; however, its added value to conventional imaging is still under debate [10,11,12,13,14,15,16] Many of these studies either require multiple follow-ups or a special radiomics platform with hundreds of rCBV parameters, limiting their applicability in everyday practice. Thus, there remains the need for an easily applicable diagnostic approach allowing for the estimation of PD probability by reviewing a few imaging parameters.

Our study aimed to estimate the value that DSC-MRI adds to conventional MR imaging for the differentiation between PD and PsP in different glioma molecular subtypes, by using a multilevel classification and a machine learning-based analysis of nine rCBV histogram parameters in two tumor VOIs.

## 2. Materials and Methods

### 2.1. Patients

**Figure 1:**
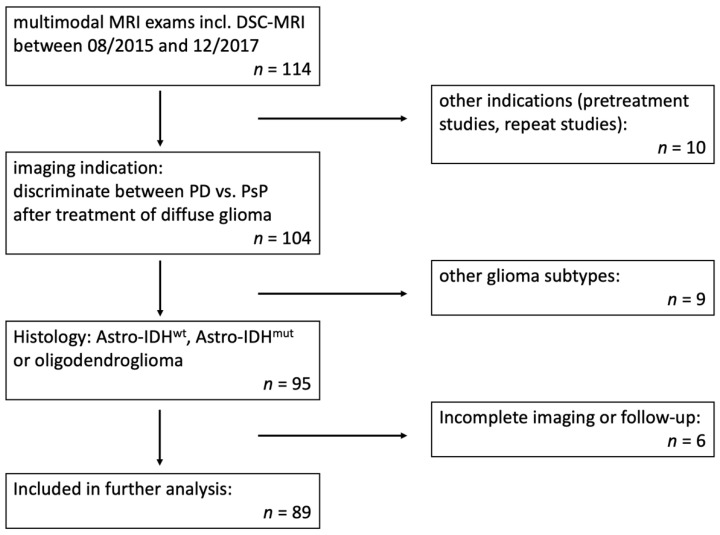
Flow chart of patient selection. Abbreviations: DSC-MRI: dynamic susceptibility contrast MRI; PD: progressive disease; PsP: pseudoprogression; Astro-IDH^wt^: isocitrate dehydrogenase wild type astrocytic tumor; Astro-IDH^mut^: astrocytic tumor with isocitrate dehydrogenase mutation.

Patients were recruited from the hospital’s imaging database in a retrospective manner; the hospital’s local review board approved the retrospective study design and waived written informed consent. All patients who received a multimodal MRI with DSC-MRI on one scanner in our institution between 08/2015 and 12/2017 were reviewed. 104 patients with the imaging indication to discriminate between PD vs. PsP after treatment were included. Subsequently, patients with previously defined histopathological diagnosis (conform to the 2016 CNS WHO criteria [17]) were reviewed and only those with astrocytic tumors (IDH wild type (Astro-IDH^wt^) or IDH mutated (Astro-IDH^mut^)) and oligodendrogliomas (IDH-mutant, ATRX-wildtype, and 1p/19q-codeletion) were included (*n* = 95). Finally, patients with incomplete imaging or missed follow-up were excluded, leading to a final patient number of 89 (Figure 1).

For the definition of PD and PsP in these patients, clinical data and histopathology (as available) were used as reference standards by retrieving the patients’ electronic health records within 6–9 months from the time of the analyzed MRI. Clinical data included the disease’s further course, clinical examination notes and follow-up imaging. Therapy regimes preceding the DSC-MRI examination (radiotherapy, chemotherapy, or immunotherapy within the previous two years) were also noted. The individual outcome for both high grade and low grade glioma was defined in accordance with the RANO [5] and if applicable, the iRANO [6] criteria [18,19,20].

Of the 89 included patients (mean age 49 ± 13 years, 39 female), 42 had Astro-IDH^wt^ (31 WHO grade IV), 27 had Astro-IDH^mut^ (5 WHO grade IV), and 20 had oligodendroglioma (Table 1). 61/89 (69%) patients had PD and 28/89 (31%) patients PsP. Histopathological confirmation was obtained in 22 of the 61 PD cases (36%).

### 2.2. MRI Examinations

MRI examinations were acquired using a 3T MRI scanner (Biograph mMR, Siemens Healthineers, Erlangen, Germany) according to the standardized brain tumor imaging protocol, including DSC-MRI [21]. DSC-MRI was performed with 3 min delay after contrast preloading with 0.25 mmol/kg Gadobutrol (Gadovist, Bayer Healthcare, Leverkusen, Germany), during the first pass of the bolus injection of 0.1 mmol/kg Gadobutrol (injection rate 3 mL/s). CBV was calculated from DSC-MRI with syngo^®^ perfusion (Siemens Healthcare, Erlangen, Germany) with an automatic definition of the arterial input function and model-based post-processing leakage correction. For rCBV histogram analysis, we used an in-house Matlab^®^-based software (Matlab 2014b, MathWorks Natick, MA, USA), as described in [7]. Two board-certified radiologists with >5 years of neuroradiology experience analyzed the images in consensus, both blinded to the outcome. The presence or absence of new or progressive contrast enhancement (CE) in tumor-suspect lesions was documented. A three-dimensional multi-slice volume of interest (VOIflair) was defined on the FLAIR images encompassing all of the tumor-suspect signal alterations but excluding necrotic and hemorrhagic areas―which would compromise the evaluation of the CBV maps―by inspecting the corresponding pre- and post-contrast T1-weighted (T1w) images. On the post-contrast T1w images, a CE VOI (if present) was defined similarly (VOIce).

After automatic distortion corrected transfer of the VOIs to the CBV maps, we normalized all CBV values to a VOI in the contralateral normal-appearing white matter, resulting in relative CBV values (rCBV) and retrieved nine parameters from the corresponding rCBV histogram: the mean, minimal, and maximal rCBV, the standard deviation, skewness, and kurtosis, as well as the 25th, 50th, and 75th percentiles.

### 2.3. Statistical Analysis

We used the Fisher exact test to assess if the presence of a CE lesions (binary non-parametric variable) could identify PD vs. PsP (binary dependent variable). Univariate logistic regression was used for the continuous variables of mean and maximal rCBV. For the combined assessment of all rCBV parameters in both VOIs we used a machine learning-based approach with a decision tree model (DTM, 18 input parameters, minimal leaf size = 2, 10-fold cross-validation), where the input parameters were the presence of a CE lesion and the corresponding nine rCBV histogram parameters (2 × 9 parameters). We included missing rCBV values as a separate category representing the absence of CE as input information. We evaluated the diagnostic imaging performance by receiver operating characteristics (ROC) analysis, including calculation of the 95%-confidence intervals. Statistical calculations were performed with the software JMP 13.0 (SAS, Cary, NC, USA) and on statpages.org. A *p*-value of less than 0.05 was considered statistically significant.

## 3. Results

### Multilevel Classification by Tumor Subtype and Imaging Parameters

The rate of PD (as defined in the Methods section using a clinical or histopathologic reference standard) was 69%. PD occurred in 86% (36/42) in the Astro-IDH^wt^ subgroup (+17% compared to the whole group), in 52% in the Astro-IDH^mut^ subgroup (−17%), and in 55% in the oligodendroglioma subgroup (−14%). Astro-IDH^wt^ tumors had a higher prevalence of PD when the MGMT promoter was unmethylated (92% compared to 81% in methylated MGMT promotors).

CE lesions were present in 63% of all patients, with the most common type being Astro-IDH^wt^ (74%), followed by Astro-IDH^mut^ (63%), and oligodendroglioma (40%). The presence of a CE lesion raised PD probability in the whole cohort to 82% vs. the initial 69% (+13%), more so in oligodendroglioma (88% vs. 55%, i.e., +33%) than in Astro-IDH^wt^ (94% vs. 86%, i.e., +8%) and in Astro-IDH^mut^ (59% vs. 52%, i.e., +7%). A significant association between the presence of a CE lesion and a higher prevalence of PD was found in Astro-IDH^wt^ (*p* = 0.0214) and oligodendroglioma (*p* = 0.0281) with the highest accuracy in Astro-IDH^wt^ (Table 2). The data of the VOIce of one patient with Astro-IDH^wt^ was technically compromised, and we only analyzed the data of the VOIflair of this patient.

The easily derivable rCBV histogram parameters mean and maximal rCBV predicted PD more accurately if there was a CE lesion, but they also yielded a reasonable accuracy in the VOIflair in the Astro-IDH^mut^ and oligodendroglioma subtypes (Table 3).

**Figure 2:**
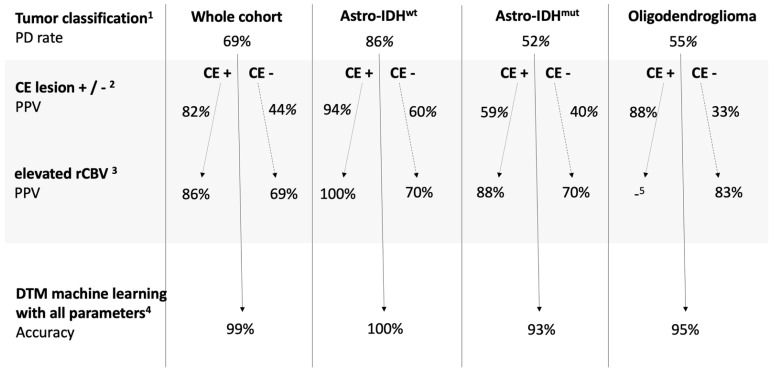
Multilevel classification for the differentiation of progressive disease vs. pseudoprogression: ^1^ The rate of progressive disease depends on the tumor type. ^2^ The positive predictive value for progressive disease is higher in the presence of a contrast-enhancing lesion in each tumor type, but not equally so. ^3^ Additional information about an elevated rCBV parameter (mean or max, above a cut-off value shown in Table 3) amends the positive predictive value especially in lesions without contrast enhancement. ^4^ The machine learning-based decision tree analysis including 2 × 9 parameters (see Methods) elevates prediction accuracy to 93% and above. ^5^ In this group, 7/8 patients had progressive disease, therefore further analysis was waived. Abbreviations: rCBV: regional cerebral blood volume; Astro-IDH^wt^: isocitrate dehydrogenase wild type astrocytic tumor; Astro-IDH^mut^: astrocytic tumor with isocitrate dehydrogenase mutation; PD: progressive disease; PsP: pseudoprogression; CE: contrast-enhancing; DTM: decision tree-based model.

The DTM, where the presence or absence of a CE lesion and nine rCBV histogram parameters were included as input information, yielded an excellent diagnostic performance with high validity in Astro-IDH^wt^ glioma and good performance with lower validity in Astro-IDH^mut^ glioma and oligodendroglioma (Table 4). The results of the multilevel classification are demonstrated in Figure 2, where each level represents the additional information resulting in the change of the probability of PD. The addition of each parameter (tumor type, CE lesion, rCBV) increases diagnostic security. The combination of all parameters in the DTM reaches the highest accuracy for detecting PD in all groups.

## 4. Discussion

With the increase of treatment options for diffuse gliomas resulting in an expansion of survival, the diagnostic dilemma of differentiating PD from PsP in the post-treatment evaluation is and will be more and more prevalent [22,23]. Clinical decision-making needs the support of neuroimaging, but using only conventional MRI its utility remains limited [5,24,25]. While DSC-MRI is increasingly available, its value in this context is still debated [26,27]. A recent review of the current strategies of glioma surveillance [28] includes a useful overview of the current literature and a pertinent discussion of the topic. It has been shown that observing rCBV changes over time is useful for PsP detection [13,29]; however, this still leaves the clinician with the need for multiple follow-ups. A radiomics approach [14] has been shown to differentiate accurately between PsP and PD; more recently, deep learning algorithms utilizing data from multiparametric MRI data were also shown to be useful in this indication [30,31]; however, as long as such a tool is not widely available for the imaging community, there remains need for an easily applicable diagnostic approach, where new suspicious imaging findings can be matched to a PD probability. The novelty of our study is that it provides a simple way to assess PD probability at the first imaging of a patient with the diagnostic question of PD vs. PsP.

The rate of PsP (31%) in our cohort was comparable with the literature [3,5]. We found a lower PsP rate (and thus a higher prevalence of PD) in Astro-IDH^wt^ (14%) compared to Astro-IDH^mut^ (48%) and oligodendroglioma (45%), in agreement with prior reports [32,33].

Also, the presence of a CE lesion had a predictive value for PD, as has been described previously [34,35]. The RANO criteria evaluate imaging changes with and without CE separately and according to other criteria for defining potential tumor progression [5]. Therefore, we included a separate no CE-lesion analysis, as previously suggested [36].

Our results confirmed previous findings of the predictive value of mean and maximal rCBV―parameters that are easily obtainable in the clinical routine. We found that rCBV performed better within the CE lesion (if present) than in the whole suspect FLAIR area, which aligns with the hypothesis that elevated rCBV reflects vessel sprouting with an impaired vascular function (concomitant with blood-brain barrier breakdown) within tumor infiltration zones in PD rather than ischemia-related scar tissue or immune-response related hypercellularity seen in PsP [27]. In the VOI comprising the whole suspect area (VOIflair), gliosis and edema may additionally intertwine with these pathophysiologic processes. In pretreatment imaging, oligodendrogliomas were shown to have higher and Astro-IDH^mut^ lower rCBV values than Astro-IDH^wt^ [37,38]. These findings were not replicated in post-treatment imaging. The DTM, combining 10 imaging parameters, had a superior performance for PD detection, similarly to the results of a radiomics based study with 310 features [14].

The multilevel classification with associated probabilities (Figure 2) might serve as a straightforward guidance tool to identify patients with PD in everyday clinical practice in addition to the RANO criteria. In summary, in Astro-IDH^wt^, PD probability is per se high and increases by the presence of a CE lesion; rCBV does not add much information. In Astro-IDH^mut^, with a chance of 52% for PsP, the presence of a CE lesion is of minor importance but an elevated rCBV speaks highly in favor of PD. In oligodendrogliomas, the presence of a CE lesion or an elevated rCBV raises the probability of PD from 45% to >80%. Cases without a CE lesion or a high-CBV-lesion are more challenging in all subgroups. However, DTM yields very high accuracy even in these groups, reflecting the added value of all rCBV histogram parameters. It is known that Astro-IDHwt have an inherently worse prognosis than Astro-IDHmut, part of which is that these tumors tend to recur rather than show PsP. It has also been shown that Astro-IDHmut glioma consistently demonstrated less aggressive imaging features than Astro-IDHwt [39]. We hypothesize that the Astro-IDHwt tumors behave a priori more malignantly, their worse prognosis resulting partly from their high PD rate, which is ascertained by the presence of a CE lesion, more so if hyperperfused. On the other hand, Astro-IDHmut tumors behave less malignantly, with a lower rate of PD, and their lesions are less prone to the disruption of the blood-brain-barrier, and even if they do enhance, this does not equate PD. However, if their lesions are hyperperfused, this indicates a more malignant behavior―thus being the scenario where functional imaging with DSC-MRI plays a relevant role in the discrimination of PD.

Our results interpretability is inherently limited by the retrospective design of the study with inhomogeneous data. We included both patients who were directly post-treatment, as well as patients with treatment more than 2 years ago; while most PsP occur in the months directly after treatment, there are reports of late onset PsP [40,41], supporting our choice to extend the time frame. Another limitation may be including only the molecular glioma characteristics regardless of WHO grades. It has been demonstrated that the survival differences between WHO grade II and grade III Astro-IDH^mut^ are not significant, while the rare WHO grade IV Astro-IDH^mut^ still have a significantly worse outcome [42]. Also, it is now the understanding that “low-grade Astro-IDH^wt^” does probably not exist and should be designated as “molecular glioblastoma” [43]. In our cohort, 73% of Astro-IDH^wt^ were WHO grade IV and 18% of Astro-IDH^mut^ were WHO grade IV tumors, which is a good representation of the generally known distributions. It has also been shown that IDH mutation status―along with other factors such as MGMT methylation, overexpression of p53 and 1p/19q codeletion―has a predictive value for PsP, with IDH-mutant tumors being more prone for PsP, which could also be reproduced in our cohort (14% PsP-rate in Astro-IDH^wt^ vs. 48% in Astro-IDH^mut^ and 45% in oligodendroglioma). MGMT methylation has also been shown to correlate with a higher occurrence of PsP [44], which could also be reproduced in our cohort; however, further subgroup analysis according to the MGMT status was omitted due to the lack of statistical power resulting from too small subgroups. These studies, however, have mainly been conducted on cohorts containing only high-grade gliomas. In summary, it might be assumed that the tumor’s molecular subtype has more to do with the probability of PsP than WHO grades. We omitted a separate or a co-analysis by WHO grades for clarity and statistical power. Another confounding factor may be our patients’ heterogeneous prior therapeutic history, also somewhat representing everyday clinical practice; subgroup analyses were not possible due to the small number of patients. Lastly, an overfitting of the DTM due to the small sample size―which was compensated for by implementing multiple cross-validation―may limit its validity.

## 5. Conclusions

Utilizing combined information about molecular tumor subtype, the presence of a contrast-enhancing lesion and multiple perfusion imaging parameters can increase diagnostic certainty in the differentiation of tumor recurrence vs. pseudoprogression in diffuse gliomas. DSC-CBV is especially helpful for the prediction of pseudoprogression in IDH-mutated glioma and oligodendroglioma.

## Figures and Tables

**Table 1:** Characteristics and preceding therapies of the patient groups.

	Astro-IDH^wt^	Astro-IDH^mut^	Oligodendroglioma
**Number of patients (*n*)**	42	27	20
Age (mean ± SD)	53 ± 14	40 ± 9	50 ± 10
Sex (female/male)	13/29	14/13	12/8
Progressive Disease (*n*)	36	14	11
**Tumor grade**			
WHO grade II (*n*)	4	13	13
WHO grade III (*n*)	7	9	7
WHO grade IV (*n*)	31	5	0
**MGMT promoter**			
Methylated (*n*)	16	11	20
Unmethylated (*n*)	25	8	0
Not known (*n*)	1	8	0
**Preceding therapy**			
Radiochemotherapy (*n*)	27	15	10
Immunotherapy (*n*)	11	4	1
None within 2 years (*n*)	4	8	9

Abbreviations: Astro-IDH^wt^: isocitrate dehydrogenase wild type astrocytic tumor; Astro-IDH^mut^: astrocytic tumor with isocitrate dehydrogenase mutation; MGMT: O6-methylguanine-DNA methyl-transferase.

**Table 2:** Predictive value of contrast enhancement for progressive disease.

	Astro-IDH^wt^	Astro-IDH^mut^	Oligodendroglioma
Prevalence of CE lesion	74% (31/42)	63% (17/27)	40% (8/20)
PD Rate in the Presence of a CE Lesion	94% (29/31)	59% (10/17)	88% (7/8)
*p*-value	0.0214 *	0.4401	0.0281 *
Accuracy	0.81 (0.66–0.91)	0.59 (0.39–0.78)	0.75 (0.51–0.91)
Sensitivity	0.83 (0.67–0.94)	0.71 (0.42–0.92)	0.63 (0.31–0.89)
Specificity	0.67 (0.22–0.96)	0.46 (0.19–0.75)	0.89 (0.52–1)

Abbreviations: CE: contrast-enhancing lesion; PD: progressive disease; Astro-IDH^wt^: isocitrate dehydrogenase wild type astrocytic tumor; Astro-IDH^mut^: astrocytic tumor with isocitrate dehydrogenase mutation. 95%-confidence intervals are shown in brackets after the parameters of diagnostic performance; *: statistical significance.

**Table 3:** Predictive value of mean and maximal rCBV in different tumor volumes for progressive disease.

Parameter	Whole Tumor (VOIflair)		Contrast-Enhancing Lesion (VOIce)		Whole Tumor (VOIflair) in Tumors without Contrast-Enhancing Lesion	
	Mean rCBV	Maximal rCBV	Mean rCBV	Maximal rCBV	Mean rCBV	Maximal rCBV
	**Astro-IDH^wt^**					
**Cut-off value ^a^**	0.81	1.7	0.77	1.18	1.03	2.06
**Accuracy**	0.64 (0.48–0.78)	0.62 (0.46–0.76)	1 (0.89–1)	1 (0.89–1)	0.7 (0.35–0.93)	0.7 (0.35–0.93)
**Sensitivity**	0.58 (0.41–0.75)	0.56 (0.38–0.72)	1 (0.88–1)	1 (0.88–1)	0.5 (0.12–0.88)	0.5 (0.12–0.88)
**Specificity**	1 (0.54–1)	1 (0.54–1)	1 (0.16–1)	1 (0.16–1)	1 (0.4–1)	1 (0.4–1)
	**Astro-IDH^mut^**					
**Cut-off value ^a^**	0.98	1.82	1.03	1.93	1.31	2.34
**Accuracy**	0.81 (0.62–0.94)	0.74 (0.54–0.89)	0.82 (0.57–0.96)	0.76 (0.5–0.93)	0.6 (0.26–0.88)	0.7 (0.35–0.93)
**Sensitivity**	0.93 (0.66–1)	0.79 (0.49–0.95)	0.9 (0.56–1)	0.7 (0.35–0.93)	1 (0.4–1)	1 (0.4–1)
**Specificity**	0.69 (0.39–0.91)	0.69 (0.39–0.91)	0.71 (0.29–0.96)	0.86 (0.42–1)	0.33 (0.04–0.78)	0.5 (0.12–0.88)
	**Oligodendroglioma**					
**Cut-off value ^a^**	0.87	1.55	no data ^b^		0.81	1.55
**Accuracy**	0.75 (0.51–0.91)	0.8 (0.56–0.94)			0.75 (0.43–0.95)	0.83 (0.52–0.98)
**Sensitivity**	0.82 (0.48–0.98)	0.91 (0.59–1)			1 (0.4–1)	1 (0.4–1)
**Specificity**	0.67 (0.3–0.93)	0.67 (0.3–0.93)			0.63 (0.25–0.91)	0.75 (0.35–0.97)

Abbreviations: rCBV: regional cerebral blood volume; Astro-IDH^wt^: isocitrate dehydrogenase wild type astrocytic tumor; Astro-IDH^mut^: astrocytic tumor with isocitrate dehydrogenase mutation. ^a^: cut-off value for identification of progressive disease in the receiver operating characteristic (ROC) analysis; ^b^: all but one of the patients with contrast-enhancing lesions in oligodendrogliomas had a progressive disease, therefore further analysis was waived for this subgroup; 95%-confidence intervals are shown in brackets after the parameters of diagnostic performance.

**Table 4:** Predictive value of the decision tree analysis using rCBV histogram parameters for progressive disease.

	Astro-IDH^wt^	Astro-IDH^mut^	Oligodendroglioma
r^2^	0.87	0.70	0.72
Cross-validated r^2^	0.97	0.32	0.55
Accuracy	1.0 (0.92–1)	0.93 (0.76–0.99)	0.95 (0.75–1)
Sensitivity	1.0 (0.9–1)	0.93 (0.66–1)	1.0 (0.72–1)
Specificity	1.0 (0.54–1)	0.92 (0.64–1)	0.89 (0.52–1)
Most Important rCBV Histogram Parameter ^a^	Mean rCBV in VOIce	Mean rCBV in VOIflair	Standard deviation of rCBV in VOIflair
Further Important rCBV Parameters	Skewness of rCBV in VOIflair	Kurtosis of rCBV in VOIflair; Minimal rCBV in VOIflair	75th percentile of rCBV in VOIflair

Abbreviations: rCBV: regional cerebral blood volume; Astro-IDH^wt^: isocitrate dehydrogenase wild type astrocytic tumor; Astro-IDH^mut^: astrocytic tumor with isocitrate dehydrogenase mutation. ^a^: Histogram parameter with the strongest influence on the decision tree analysis (DTM). 95%-confidence intervals are shown in brackets after the parameters of diagnostic performance.

## Data Availability

All data related to this study can be provided from the authors upon request.

## Abbreviations

Astro-IDH^mut^	astrocytic tumor with isocitrate dehydrogenase mutation
Astro-IDH^wt^	isocitrate dehydrogenase wildtype astrocytic tumor
CBV	cerebral blood volume
CE	contrast enhancement
CNS	central nervous system
DSC-MRI	dynamic susceptibility perfusion imaging
DTM	decision tree model
FLAIR	fluid-attenuated inversion recovery
IDH	isocitrate dehydrogenase
MGMT	O6-methylguanine-DNA methyl-transferase
MRI	magnetic resonance imaging
PD	progressive disease
PPV	positive predictive value
PsP	pseudoprogression
RANO	Response Assessment in Neuro-Oncology
ROC	receiver operating characteristics
rCBV	relative cerebral blood volume
SD	standard deviation
VOIce	contrast-enhancing disease-specific volume of interest
VOIflair	volume of interest with suspicious disease-specific signal alterations in the FLAIR
WHO	World Health Organization
